# Genome‐wide binding analysis of AtGNC and AtCGA1 demonstrates their cross‐regulation and common and specific functions

**DOI:** 10.1002/pld3.16

**Published:** 2017-10-16

**Authors:** Zhenhua Xu, José A. Casaretto, Yong‐Mei Bi, Steven J. Rothstein

**Affiliations:** ^1^ Department of Molecular and Cellular Biology University of Guelph Guelph ON Canada

**Keywords:** *Arabidopsis thaliana*, ChIP‐seq, GATA transcription factors, plant development, plant greening

## Abstract

GATA transcription factors are involved in multiple processes in plant growth and development. Two GATA factors, *NITRATE‐INDUCIBLE*,*CARBON METABOLISM‐INVOLVED* (*GNC*) and *CYTOKININ‐RESPONSIVE GATA FACTOR 1* (*CGA1*, also named *GNL*), are important regulators in greening, flowering, senescence, and hormone signaling. However, their direct target genes related to these biological processes are poorly characterized. Here, GNC and CGA1 are shown to be transcription activators and by using chromatin immunoprecipitation sequencing (ChIP‐seq), 1475 and 638 genes are identified to be associated with GNC and CGA1 binding, respectively. Enrichment of diverse motifs in the peak binding regions for GNC and CGA1 suggests the possibility that these two transcription factors also interact with other transcription factors and in addition genes coding for DNA‐binding proteins are highly enriched among GNC‐ and CGA1‐associated genes. Despite the fact that these two GATA factors are known to share a large portion of co‐expressed genes, our analysis revealed a low percentage of overlapping binding‐associated genes for these two homologues. This suggests a possible cross‐regulation between these, which is verified using ChIP‐qPCR. The common and specific biological processes regulated by GNC and CGA1 also support this notion. Functional analysis of the binding‐associated genes revealed that those encoding transcription factors, E3 ligase, as well as genes with roles in plant development are highly enriched, indicating that GNC and CGA1 mediate complex genetic networks in regulating different aspects of plant growth and development.

## 1 INTRODUCTION

GATA transcription factors are present across eukaryotic species and are characterized by a distinctive and conserved type IV zinc finger DNA‐binding domain, CX_2_CX_17–20_CX_2_C, which specifically recognizes the consensus DNA sequence WGATAR (W = T or A; R = G or A) (Lowry & Atchley, 2000). A recent study revealed that, in a slight variance from what has been described in animals, plant GATA transcription factors preferentially recognize and bind GATC rather than the GATA motif, and the two bases in the middle (AT) are more conserved than the flanking sequence of the binding core across the GATA family (Weirauch et al., 2014).

The existence of GATA transcription factors in plants was exposed by the GATA motifs present in the promoters of light and circadian responsive genes (Arguello‐Astorga & Herrera‐Estrella, 1998), suggesting the possible function of GATA factors in light regulation. In plants, GATA factors function in various processes, including wound defense (Sugimoto, Takeda, & Hirochika, 2003), shoot apical meristem and flower development (Zhao et al., 2004), seed germination (Liu, Koizuka, Martin, & Nonogaki, 2005), light response (Manfield, Devlin, Jen, Westhead, & Gilmartin, 2007), organogenesis (Wang et al., 2009) as well as integration of light and BR signaling pathways in regulating seedling photomorphogenesis (Luo et al., 2010).

The most studied GATA factors are two homologues named *NITRATE‐INDUCIBLE, CARBON METABOLISM‐INVOLVED* (*GNC*) and *CYTOKININ‐RESPONSIVE GATA FACTOR 1* (*CGA1*, also named *GNL*, for *GNC‐LIKE*). The *GNC* gene was first identified in *Arabidopsis* by screening the response of a set of mutants in the GATA gene family to nitrogen limitation conditions. The *gnc* mutant accumulates less chlorophyll than WT under both nitrogen sufficient and limitation conditions (Bi et al., 2005). Nitrogen induces the expression of *GNC*, which is involved in nitrogen and carbon metabolism possibly by regulating the expression of *Glutamate Synthase* (*GLU1/Fd‐GOGAT*) (Bi et al., 2005; Hudson et al., 2011). *CGA1* was characterized in *Arabidopsis* by its rapid induction by cytokinin as well as light. The significantly reduced expression of *CGA1* in mutants of the cytokinin receptor *ahk2/3* and of the red light receptor *phyA/B* indicated its involvement in cytokinin and light signaling pathway (Naito, Kiba, Koizumi, Yamashino, & Mizuno, 2007). *GNC* and *CGA1* showed similar expression patterns and loss of function mutants displayed a similar phenotype with additive phenotypes observed in the *gnc cga1* double mutant (Manfield et al., 2007; Mara & Irish, 2008). These two GATA factors were reported to be involved in multiple processes and signaling pathways in *Arabidopsis*. For example, they function downstream of the floral homeotic genes controlling floral organogenesis (Mara & Irish, 2008) and act as downstream effectors of *HAN* in regulating floral organ specification (Zhang et al., 2013).

The most prominent functions of *GNC* and *CGA1* are revealed by the phenotypes of the *gnc cga1* double mutant which displays reduced chlorophyll, early flowering and early senescence (Bi et al., 2005; Chiang et al., 2012; Richter, Behringer, Müller, & Schwechheimer, 2010). Over‐expression of *GNC* or *CGA1* in *Arabidopsis* results in ectopic accumulation of chloroplasts even in epidermis and roots, where chloroplasts are usually not found (Chiang et al., 2012). In addition, the development of chloroplasts is enhanced in *GNC* or *CGA1* over‐expression lines even in the dark, which is the opposite to the lower number of chloroplasts observed in the *gnc cga1* double mutant (Chiang et al., 2012). Along with modifications in plant architecture, similar alterations in chlorophyll content and chloroplast number were also observed in rice *OsCGA1* transgenic lines (Hudson et al., 2013). In *Arabidopsis*,* GNC* and *CGA1* were identified to be involved in a cross‐regulation mechanism with *SOC1*, acting upstream of *SOC1* to control flowering time as well as greening. In turn, *GNC* and *CGA1* are also targets of *SOC1* in the response to cold stress (Richter, Bastakis, & Schwechheimer, 2013a).

Recent studies in *Arabidopsis* revealed that *GNC* and *CGA1* are integrated with hormone signaling pathways involving gibberellic acid (GA) and auxin (Richter, Behringer, Zourelidou, & Schwechheimer, 2013b; Richter et al., 2010). Over‐expression lines of *GNC* or *CGA1* resemble the phenotype observed in the GA signaling mutant *ga1*, and a mutation in *GNC* or *CGA1* is able to suppress the *ga1* phenotype, with additive suppression in the *gnc cga1* double mutant (Richter et al., 2010). *GNC* and *CGA1* were shown to function downstream from the DELLA protein and PIF transcription factors (Richter et al., 2010). In addition, an auxin response factor mutant, *arf2*, resembles the phenotype of *GNC* or *CGA1* over‐expressors and the *gnc cga1* double mutant could suppress the *arf2* phenotype (Richter et al., 2013b), indicating an integration of *GNC* and *CGA1* in the auxin signaling pathway. Furthermore, constitutive activation of GA signaling is able to suppress the *arf2* phenotype by repressing *GNC* and *CGA1*, which are downstream targets of *ARF2*. All these observations strongly indicate the convergent regulation of auxin and GA signals on *GNC* and *CGA1* (Richter et al., 2013b). Other recent studies of the *Arabidopsis* LLM‐domain‐containing class B‐GATA factors (B‐GATAs), including *GNC* and *CGA1*, revealed their further and partially redundant functions in greening, hypocotyl elongation, and cytokinin‐regulated development (Behringer, Bastakis, Ranftl, Mayer, & Schwechheimer, 2014; Ranftl, Bastakis, Klermund, & Schwechheimer, 2016). They have also been suggested to act downstream of light signaling pathways to promote stomatal development in hypocotyls (Klermund et al., 2016).

Despite the involvement of GNC and CGA1 in multiple processes and pathways affecting plant growth and development, the downstream target genes of these two GATA factors have been barely characterized. To further understand the multiple functions of the *Arabidopsis GNC* and *CGA1* genes in transcriptional regulation, we have identified their possible target genes using a ChIP‐sequencing (ChIP‐seq) approach. Our findings suggest that GNC and CGA1 are associated with complex genetic networks involved in the regulation of developmental processes. Further, we present evidence that GNC and CGA1 cross‐regulate each other which accounts for their overlapping and diverse physiological roles.

## 2 MATERIALS AND METHODS

### 2.1 Plant materials and growth conditions

The *Arabidopsis myc‐CGA1* and *myc‐GNC* overexpression lines with c‐myc epitope tag were previously reported (Hudson et al., 2011). T‐DNA insertion lines for *AtGNC* (SALK_001778) and *AtCGA1* (SALK_003995) were obtained from the *Arabidopsis* Biological Resource Center (Bi et al., 2005). The *gnc cga1* double mutant was generated by crossing the individual mutants.

*Arabidopsis* plants were planted in Sunshine LA4 soil mix (Sun Gro Horticulture Ltd.) and grown in growth chambers (22°C day/18°C night, 16 hr light/8 hr dark, 150 μmol m^−2^ s^−1^ light density, 60% humidity).

### 2.2 Transcriptional activity assay in protoplasts

The plasmid containing the GAL4‐LUC reporter and *35S::*GAL4BD‐VP16 constructs was requested from Dr. Shouyi Chen (Hao et al., 2011). To generate an empty construct and a *35S::*GAL4BD construct as negative controls, the *35S::*GAL4BD‐VP16 plasmid was digested by *Sac*I+*Sma*I and *Sal*I+*Kpn*I to remove the GAL4BD+VP16 or VP16, respectively, and followed by re‐ligation after filling‐in the overhangs. To generate the *35S::*GAL4BD‐AtGNC and *35S::*GAL4BD‐AtCGA1 constructs, the full‐length *GNC* and *CGA1* cDNAs were amplified from Col‐0 *Arabidopsis* wild‐type plants using the primer sets listed in the Table S2 and were cloned into the *Apa*I and *Kpn*I sites of the *35S::*GAL4BD vector. The renilla luciferase gene was amplified from pRL vector (Promega) and cloned into *Sma*I and *Not*I sites of pUC18 vector to generate the *35S::*RLUC construct as the internal control.

Protoplasts were isolated from *Arabidopsis* leaves according to a previously reported method (Wu et al., 2009). Protoplast transfection and the following luciferase assay were adapted from a previous protocol (Yoo, Cho, & Sheen, 2007) and the instructions for the Dual‐Luciferase^®^ Reporter Assay System (Promega). A total of 15 μg of plasmids containing the effector (6.9 μg), reporter (6.9 μg) and reference (1.2 μg) constructs were transfected with ~2 × 10^4^ protoplasts. Plasmids containing empty (or *35S::*GAL4BD), GAL4‐LUC and *35S::*RLUC constructs were used as negative controls. Plasmids containing *35S::*GAL4BD‐VP16, GAL4‐LUC, and *35S::*RLUC constructs were used as transactivation controls. Firefly and renilla luciferase intensity was quantified using Omega Luminometer (BGM Labtech) to calculate the relative LUC intensity.

### 2.3 ChIP‐seq

The *Arabidopsis myc‐CGA1* and *myc‐GNC* overexpression lines were used for the ChIP‐seq experiment. ChIP‐DNA was prepared from the leaves of 3‐week‐old *myc*‐tagged overexpression plants using the protocols described in (Gendrel, Lippman, Martienssen, & Colot, 2005). Briefly, freshly harvested leaves were cross‐linked in 1% formaldehyde under vacuum. The chromatin was extracted, sonicated (10% sonicated chromatin was saved as INPUT sample without incubating with anti‐*myc* antibody), and the DNA was immunoprecipitated by anti‐*myc* antibody (Millipore) as the IP sample. Both IP and INPUT DNA were purified, and over 15 ng of each DNA from two biological replicates of each genotype was sent for library construction and high‐throughput sequencing using a Hi‐Seq 2500 sequencer (Clinical Genomics Center, Mount Sinai Hospital, Toronto, Canada).

### 2.4 ChIP‐seq data analysis

Raw sequencing reads were trimmed, and then, all the reads were mapped to the *Arabidopsis* genome (TAIR 10 from https://www.Arabidopsis.org) using Bowtie2 software (Langmead & Salzberg, 2012) (http://bowtie-bio.sourceforge.net/bowtie2/index.shtml) under the default option except with ‐N 1 — no‐unal, which means the mapping process allows one mismatch between each read and the genome, and the final report does not include those reads, which are not mapped to the genome. Then multiple loci mapping reads were removed using the grep command by filtering out the reads with the tab of XS:i:, which only presents when a read matches multiple locations on the genome. Peak calling was performed using Model‐based Analysis for ChIP‐seq package MACS 1.4 (Zhang et al., 2008) (http://liulab.dfci.harvard.edu/MACS/index.html). Some default MACS options were adjusted: Effective genome size was set as 1.2e+8, which is the proximate *Arabidopsis* genome size; band width was optimized to make sure a well‐shaped peak shift model is generated and ‐w ‐S arguments were used to allow MACS program generating a single wiggle file including each chromosome for peak visualization. The *plotDistToTSS* function in the R package ChIPSeeker was used to plot the distance of the peaks around the transcription start site (TSS) (Yu, Wang, & He, 2015). Then, the .bed file generated by MACS was submitted to ChIPSeek, which integrates the HOMER package for peak annotation (Chen et al., 2014) (http://chipseek.cgu.edu.tw/index_show.py). The peak distribution was summarized based on the peak location, and only promoter‐related peaks were saved for further analysis. For motif analysis, each peak summit was extracted and ±250‐bp sequence around it was used for motif search in ChIPSeek. CIS‐BP database (http://cisbp.ccbr.utoronto.ca/index.php) was also retrieved for *cis*‐motifs of *Arabidopsis* GATA family (Weirauch et al., 2014). The R function “matchPattern” in the R package “BSgenome” (Pagès, 2016) was applied to identify all the GATA‐like motifs (GATA/C) in the complete *Arabidopsis* genome.

### 2.5 Gene ontology and pathway analysis

Gene ontology (GO) enrichment analysis was performed using agriGO (Du, Zhou, Ling, Zhang, & Su, 2010) (http://bioinfo.cau.edu.cn/agriGO/). The GO tool on the TAIR webpage (https://www.Arabidopsis.org/tools/bulk/go/index.jsp) was also used to functionally categorize the number of GNC‐ or CGA1‐associated genes to each GO term. Within each dataset, the relative abundance of genes associated with each GO term was compared to that in the whole genome and the enrichment or underrepresentation of each GO term was analyzed by calculating the *p*‐value using a hypergeometric test. Different pathway analysis was conducted using MapMan software (Thimm et al., 2004) (http://mapman.gabipd.org/web/guest/mapman).

### 2.6 ChIP‐qPCR analysis

The prepared ChIP‐DNA from *myc*‐AtGNC and *myc*‐AtCGA1 overexpression lines was applied for qPCR analysis using respective primers (Table S2). Primers were designed by Primer Express 3.0 (Applied Biosystems) around the peak summit of each gene based on the ChIP‐seq result. A pair of primers designed for the *Actin 7* promoter was used as negative control for ChIP‐qPCR. Anti‐IgG antibody was used independently in the ChIP‐DNA preparation and the resulting DNA served as a negative control for the ChIP assay. ChIP‐qPCR was performed in triplicate for each sample using PerfeCTa SYBR Green Fast Mix (Quanta Biosciences) on ABI 7300 Real‐Time PCR System (Applied Biosystems). The expression level was normalized against the INPUT sample, and fold enrichment was calculated and presented as % INPUT according to (Lin, Tirichine, & Bowler, 2012).

### 2.7 Gene expression analysis

Total RNA from three biological replicates of each sample was isolated using the Trizol Reagent (Life Technologies) according to the user manual. RNA was treated by DNase I (Promega), and then, 1 μg of DNA‐free total RNA was used for cDNA synthesis with qScript™ cDNA SuperMix (Quanta Biosciences) according to the manufacturer's instructions. The cDNA was diluted 10 times after the reverse transcription reaction, and 1 μl was used in real‐time PCR. Primers (Table S2) were designed using Primer Express 3.0 (Applied Biosystems), and real‐time PCR was performed using PerfeCta SYBR Green FastMix (Quanta Biosciences) on an ABI 7300 Real‐time PCR System (Applied Biosystems). PCRs were performed in triplicate for each biological replication. Expression levels were normalized against *Arabidopsis UBC21*, and relative expression was calculated using the 2^−ΔΔCt^ method (Livak & Schmittgen, 2001). All the expression data were presented as fold change value and were transformed to log2 values for statistical analysis.

### 2.8 Statistical analysis

Student's *t* test and one‐way ANOVA analyses were performed using the SPSS software (IBM), and the LSD method was used for multiple comparisons.

### 2.9 Accession numbers

Sequence data from this article can be found in the EMBL/GenBank data libraries under accession numbers: AT5G56860 (*AtGNC*), AT4G26150 (*AtCGA1*). Raw sequencing and processed ChIP‐seq data have been deposited to NCBI's GEO and can be found under the accession number: GSE97499.

## 3 RESULTS

### 3.1 *Arabidopsis* GNC and CGA1 function as transcription activators

**Figure 1:**
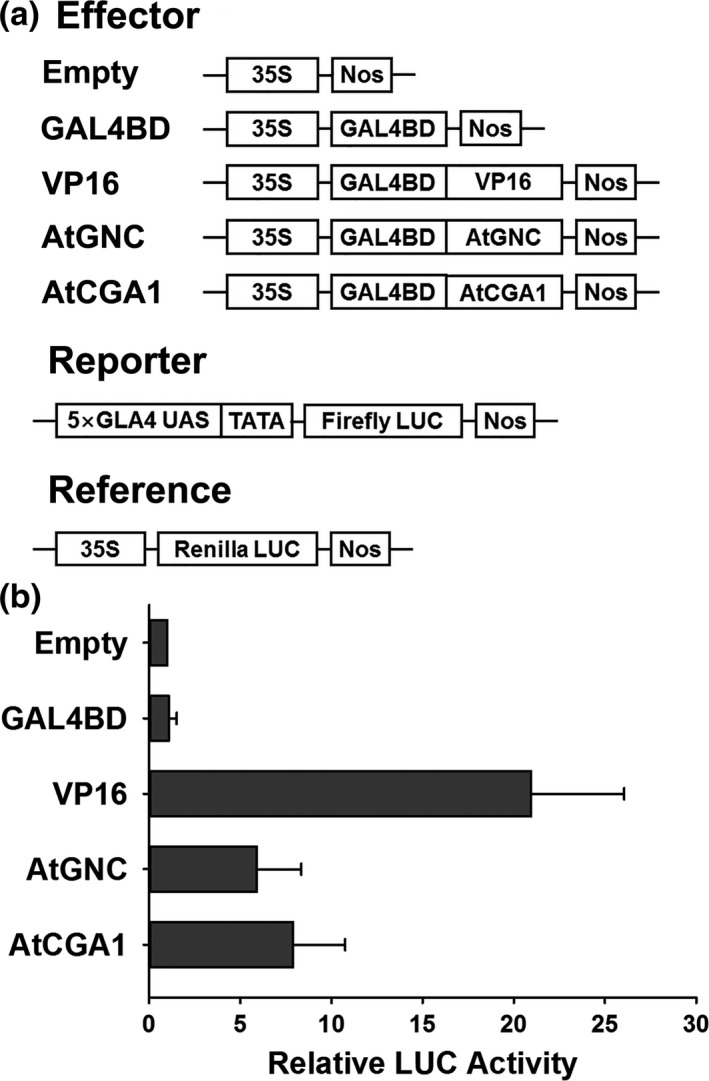
*Arabidopsis* GNC and CGA1 can function as transcriptional activators. (a) Scheme of the effector, reporter, and reference constructs used in the transactivation assay. (b) Relative firefly luciferase activity when reporter and reference constructs were co‐transfected with different effectors into *Arabidopsis* protoplasts

To confirm the transcription activity of *Arabidopsis* GNC and CGA1, a dual‐luciferase reporter system was used in *Arabidopsis* protoplasts. As a positive control for transactivation, the transactivation domain of VP16 was fused to the GAL4 DNA‐binding domain (GAL4BD) under the control of the CaMV 35S promoter. The other effectors were modified based on the GAL4BD‐VP16 effector (Figure 1a). The expression of the firefly luciferase reporter gene (LUC) was driven by five copies of the GAL4 binding element with a minimal 35S (TATA box) promoter (Figure 1a). Expression of the renilla luciferase gene was driven by the 35S promoter as an internal control (Figure 1a). Compared to the empty and GAL4BD negative controls and the VP16 transactivation effector, GNC and CGA1 strongly activated the expression of the LUC reporter gene (Figure 1b), suggesting that *Arabidopsis* GNC and CGA1 function as transcription activators.

### 3.2 Determination of AtGNC and AtCGA1 binding sites by ChIP‐seq

To further understand the regulatory mechanism of GNC and CGA1 for multiple functions, a ChIP‐seq analysis was carried out to identify possible target genes of these two transcription factors in vivo. ChIP‐DNA as well as INPUT DNA from whole rosette leaves of *myc*‐AtGNC and *myc*‐AtCGA1 overexpression lines (two biological replicates) was used in this assay (Fig. S1). Model‐based Analysis of ChIP‐seq was used to identify the enriched GNC or CGA1 binding sites (Fig. S2).

**Figure 2:**
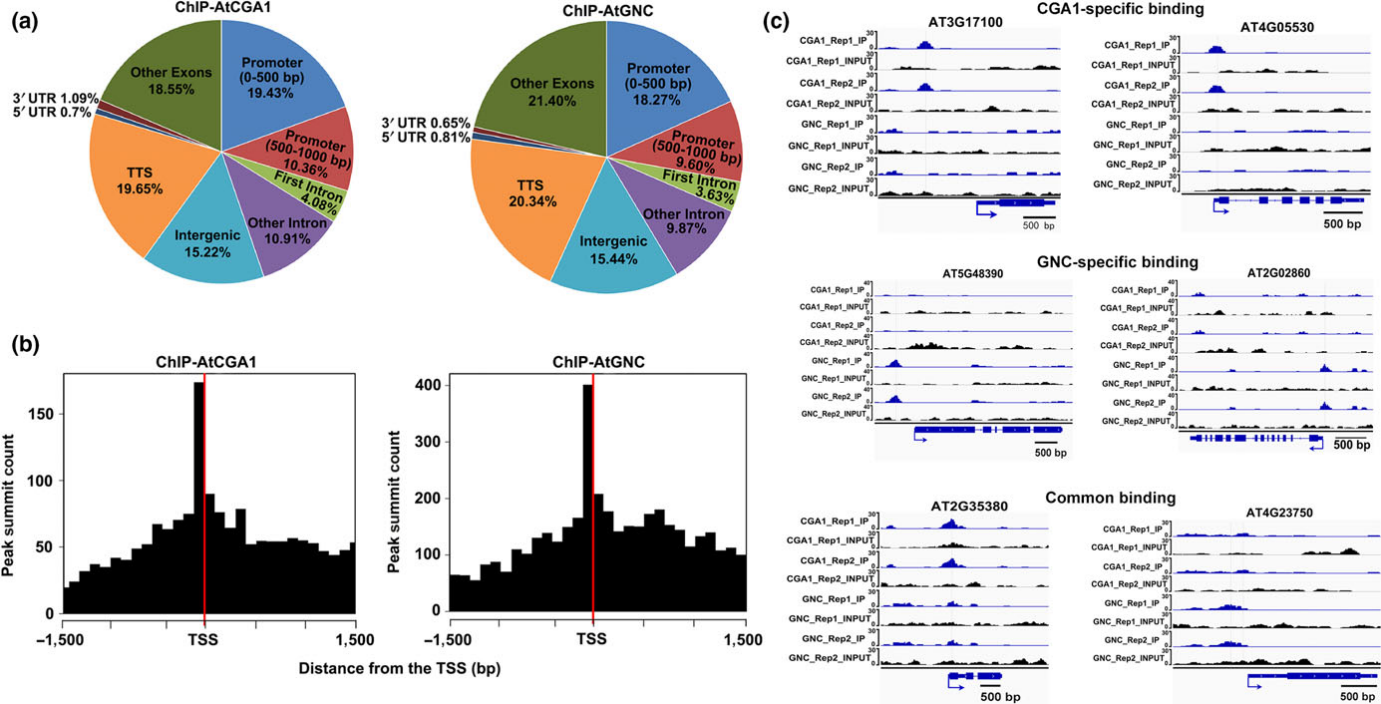
Genomewide binding sites of *Arabidopsis* GNC and CGA1. (a) Distribution of GNC and CGA1 binding sites. (b) GNC and CGA1 binding sites are highly enriched in the first 200 bp of the promoter region upstream of the transcription start site (TSS). (c) Representative peaks related to CGA1‐specific binding, GNC‐specific binding, and CGA1 and GNC common binding

The detected binding sites were annotated against the *Arabidopsis* TAIR 10 genome (Data S1). Around 30% of all binding peaks for both genes distributed along the genome were highly enriched in the first 1 kb of the promoter region, which was the most enriched region compared with other genome locations (Figure 2a). Further analysis of the binding peaks around the transcription start site (TSS) revealed that the binding sites are mainly enriched in the first 200 bp directly upstream of the TSS in the promoter regions (Figure 2b; Fig. S1d). The binding profiles of GNC and CGA1 also showed similar distribution on the genome (Figure 2a,b). For both transcription factors, the representative binding peaks located in the promoter region of target genes with different fold enrichment (FE) against INPUT are shown in Figure 2c. All the binding profiles were consistent with the fact that GNC and CGA1 function as transcription factors.

### 3.3 Identification of GNC‐ or CGA1‐associated genes reveals that these two transcription factors are cross‐regulated

**Figure 3:**
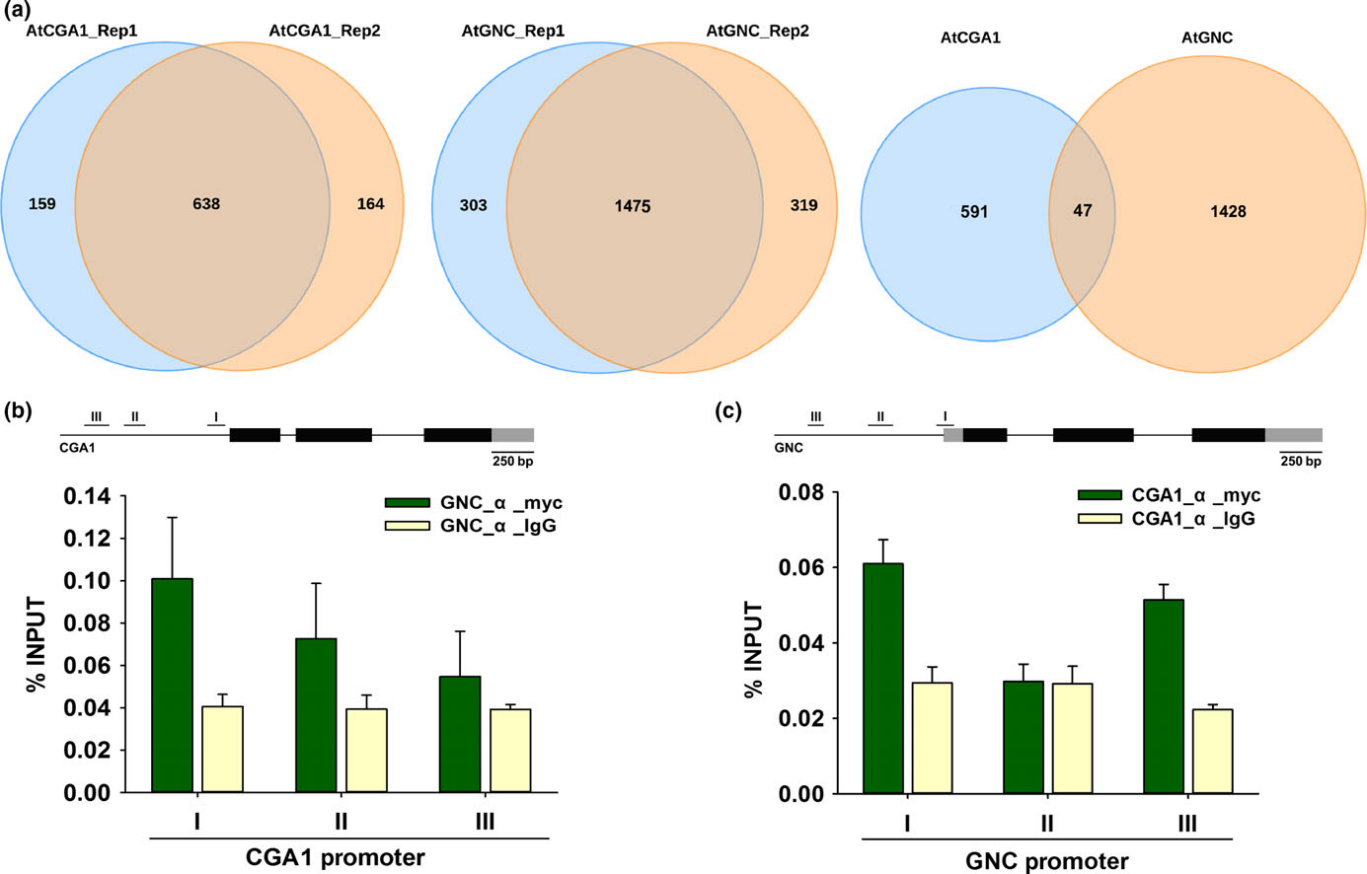
GNC and CGA1 are cross‐regulated. (a) The overlapping genes between the two replicates of GNC or CGA1 binding peaks were considered as the binding‐associated genes, which were used to identify the shared targets between GNC and CGA1. (b) Structure of the *CGA1* gene within the 1 kb promoter and ChIP‐qPCR confirmed the binding of GNC on different regions of the *CGA1* promoter. I, II, and III were three qPCR amplification regions. Data represented mean ± SD (*n* = 3). (c) Structure of the *GNC* gene within 1 kb promoter and ChIP‐qPCR confirmed the binding of CGA1 at different regions of the *GNC* promoter. I, II, and III were the three qPCR amplification regions. Data represented mean ± SD (*n* = 3)

According to the criteria of the peak annotation program, genes that had binding peaks within the positions −1 kb to +100 bp from the TSS were considered as GNA or CGA1 binding‐associated genes and were selected for further analysis. Within the binding profiles of the two replicates of GNC or CGA1 ChIP‐seq datasets, a high percentage of overlapping genes (around 80%) were found (Figure 3a). This indicated that the quality of the data generated from the biological replicates were representative, which was further validated by chromatin immunoprecipitation quantitative PCR (ChIP‐qPCR) to quantify nine random selected genes present in both of the GNC and CGA1 ChIP‐seq datasets (Fig. S3). The overlapped genes between the replicates of GNC or CGA1 binding profiles, 1475 and 638, respectively, were designated as GNC‐ or CGA1‐associated genes and were used for further functional analysis (Figure 3a; Data S2). It was surprising to find a very low percentage of overlap between GNC‐ and CGA1‐associated genes (Figure 3a; Data S2). Due to the fact that these two transcription factors share a high percentage (~70%) of co‐expressed genes according to the ATTED‐II database (Aoki, Okamura, Tadaka, Kinoshita, & Obayashi, 2016) (Data S6), we propose that GNC and CGA1 could be cross‐regulated. Primers were designed for three regions of the GNC and CGA1 promoter regions (1 kb upstream of the TSS) harboring multiple GATA/C‐like motifs. The results of the ChIP‐qPCR confirmed our hypothesis that GNC can bind the promoter of *CGA1* (Figure 3b) and that CGA1 can also bind the promoter of *GNC* (Figure 3c). Expression analysis revealed that CGA1 was up‐regulated about 10‐fold in the *gnc* mutant (Fig. S8a) and GNC was up‐regulated about 1.6‐fold in the *cga1* mutant (Fig. S8b), suggesting the existence of a more complex compensation mechanism between GNC and CGA1 in regulating gene expression.

### 3.4 Identification of GNC and CGA1 binding Motifs

**Figure 4:**
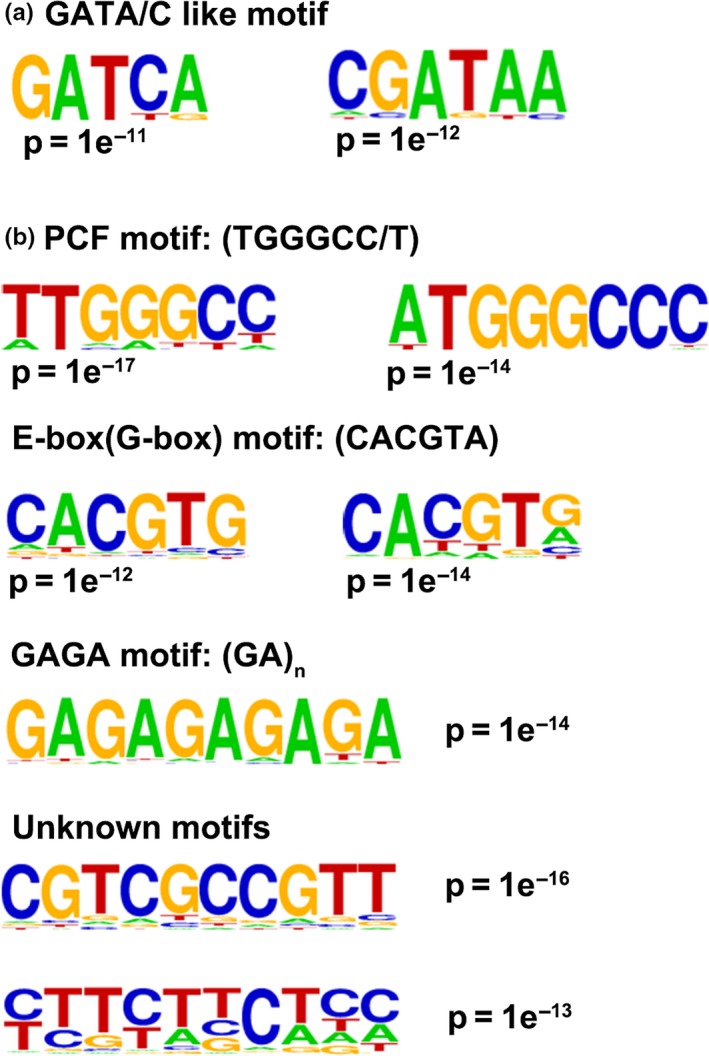
*cis*‐motifs enriched in the GNC and CGA1 binding peaks. (a) GATA/C‐like motifs enriched in the GNC and CGA1 binding peaks. (b) Non‐GATA/C‐like motifs enriched in the GNC and CGA1 binding peaks

To investigate the binding motifs for GNC and CGA1, the ±250‐bp sequence flanking the ChIP‐seq peak summits was extracted and used as input data for motif search with the ChIPSeek program. Common or similar motifs present in both GNC and CGA1 binding peaks were identified as binding motifs for these two transcription factors. Similar GATA/C‐like motifs to those previously reported for GATA factors (Lowry & Atchley, 2000; Weirauch et al., 2014) were also enriched in the binding peaks for GNC and CGA1 (Figure 4a). However, the GATA/C‐like motifs were not highly represented compared to other motifs also found in this analysis (Figure 4), probably because the frequency of the presence of the GATA and GATC sequences, in the whole genome, occurs every 288 bp and 274 bp, respectively (Table S1). Information about DNA‐binding motifs for most members of the *Arabidopsis* GATA family (Weirauch et al., 2014) was also gathered from the CIS‐BP database. Direct evidence from the protein binding microarray assay revealed binding motifs for almost half of the *Arabidopsis* GATA factors (Fig. S4a), whereas the binding motifs for other members were inferred based on the identity of the DNA‐binding domain (DBD) (Fig. S4b). The analysis showed that the binding motif for GNC and CGA1 is likely to be similar to that of GATA15 (Fig. S4).

**Figure 5:**
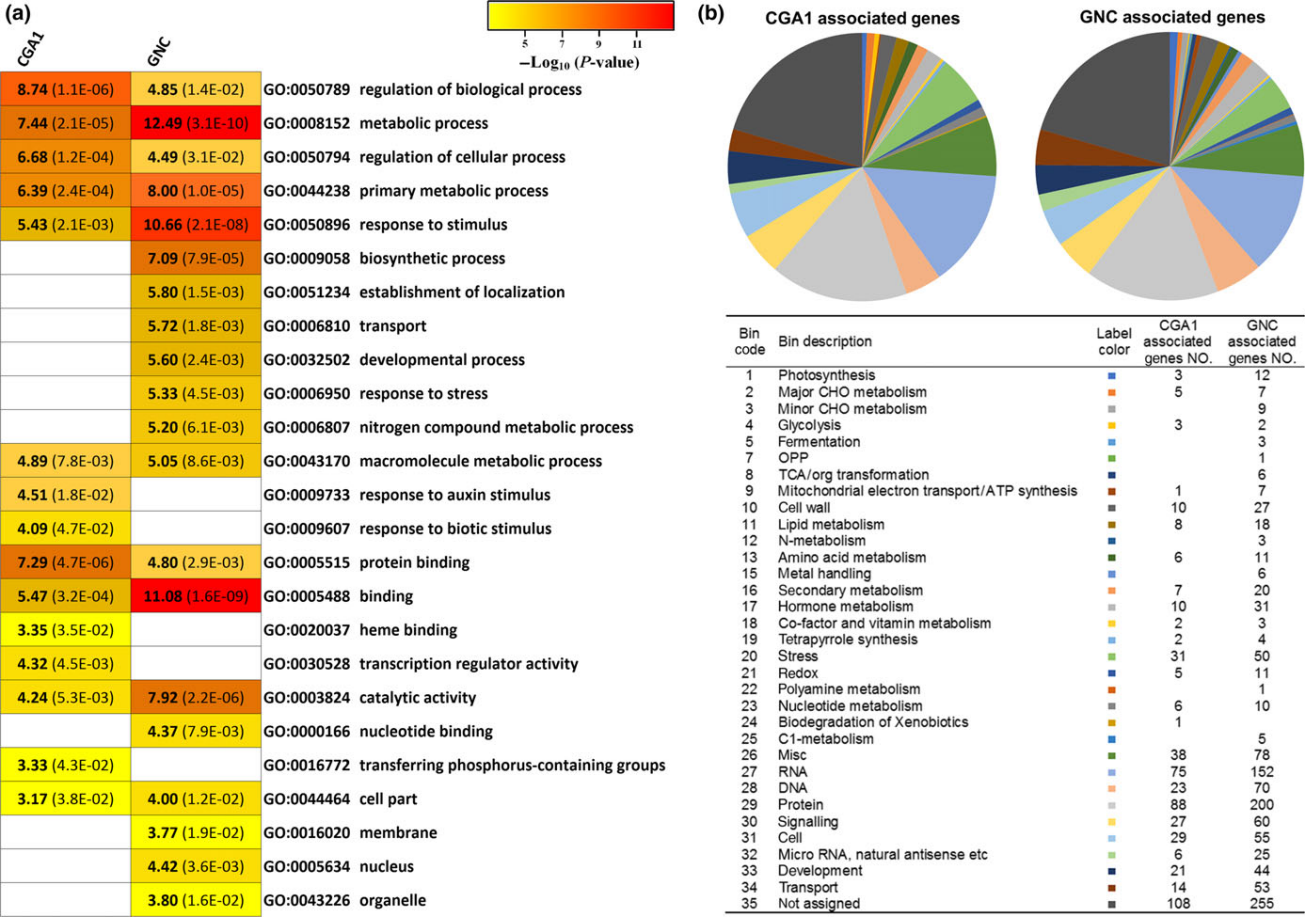
Functional characterization of GNC‐ and CGA1‐associated genes. (a) GO terms enriched in the GNC‐ and/or CGA1‐associated genes. (b) Pathway analysis showed the number of GNC‐ and CGA1‐associated genes in different biological processes and pathways

Several other non‐GATA/C motifs were also identified in the ChIP binding peaks, such as PCF, E‐box, and GAGA motifs as well as other unknown motifs, implying that there may be multiple transcription factors involved in regulating these genes (Figure 4b). In this regard, transcription factors were also highly enriched among the GNC‐ and CGA1‐associated genes, such as PCF motif‐binding TCP factors as well as E‐box motif‐binding bHLH factors (Data S2 and S5). This suggests that GNC and CGA1 regulate the expression of these transcription factors which concurs with the fact that the *protein binding* and *transcription factor activity* in the GO analysis were overrepresented among the GNC‐ and CGA1‐associated genes (Figures 5, S5).

### 3.5 Common and specific roles of GNC and CGA1 in regulating various processes of plant growth and development

To further understand the involvement of GNC and CGA1 in *Arabidopsis* growth and development, functional analysis for the GNC‐ and CGA1‐associated genes was performed. Gene ontology (GO) and function classification revealed that GNC and CGA1 regulate multiple processes of plant growth and development (Figure 5). GO terms enriched for GNC or CGA1 putative targets exhibited common and specific functions. For example, protein binding (GO:0005515) was enriched in both GNC‐ and CGA1‐associated genes whereas nitrogen compound metabolic process (GO:0006807) was only enriched in GNC but not in CGA1‐associated genes (Figure 5a), indicating that GNC role is probably more specific than CGA1 in nitrogen metabolism, which is consistent with its known function (Bi et al., 2005). Classification of the main GO categories also exhibits commonalities and specific functions (Fig. S5; Data S3). For instance, the GO term *transcription factor activity* under the molecular function category and the GO term *signal transduction* under biological process category were significantly enriched in both GNC‐ and CGA1‐associated genes, whereas some terms were only enriched in either GNC‐ or CGA1‐associated genes such as *chloroplast* under the cellular component category (Fig. S5). Moreover, some GO terms such as *transporter activity* under the molecular function category were enriched in GNC‐associated genes, whereas it was underrepresented in CGA1‐associated genes (Fig. S5). This functional specificity of these two transcription factors provides further evidence for the possibility of a cross‐regulation between them.

In addition, the Mapman program was used to assign GNC‐ or CGA1‐associated genes to a specific functional or metabolic pathway. The results also revealed that GNC and CGA1 are involved in multiple biological pathways (Figures 5b, S6; Data S4, S5). GNC‐associated genes were generally more enriched in different functions or pathways compared to those of CGA1‐associated genes (Figures 3a, 5b, S5; Data S4). Protein‐related processes have the most genes assigned, accounting for ~16% and ~17% of GNC‐ and CGA1‐associated genes, respectively, following by the RNA‐related process, which accounts for ~12% and ~14% of GNC‐ and CGA1‐associated genes, respectively (Figure 5b). This suggests that GNC and CGA1 may play more important roles in processes such as protein modification and degradation as well as in transcriptional regulation. About 5% of the GNC‐ or CGA1‐associated genes were involved in signaling pathways, suggesting that GNC and CGA1, as transcription factors, control genes in different cell signaling transduction cascades (Figure 5b). About 4% of GNC‐associated genes and ~6% of CGA1‐associated genes were involved in stress response, especially biotic stress (Figure 5b, S5; Data S6), suggesting that GNC and CGA1 may function in biotic stress response. In addition, ~20% of both GNC‐ or CGA1‐associated genes had no functional pathways assigned, indicating that these two transcription factors may also play roles in other undefined processes (Figure 5b).

### 3.6 GNC‐ and CGA1‐associated genes are involved in protein binding, modification and degradation

GO analysis showed that *protein binding* was highly enriched in both GNC and CGA1 binding‐associated genes compared with the corresponding frequency in the whole genome (Fig. S5). The pathway analysis assigned about 16% of the target gene sets of GNC and CGA1 protein‐related processes, which represents the majority of genes compared to other biological pathways (Figure 5b; Data S4). These target genes were involved in protein synthesis, targeting, posttranslational modification, and degradation (Data S4). In addition, the GO term *protein metabolism* was not overrepresented among the GNC‐ and CGA1‐associated genes when compared to that in the whole genome (Fig. S5), indicating that GNC and CGA1 may not be directly involved in protein anabolism or catabolism. Interestingly, genes encoding proteins involved in ubiquitination‐mediated protein degradation, more specifically, the E3 ubiquitin ligase (Data S4, S5), were overrepresented, suggesting that GNC and CGA1 may participate in the quality control and homeostasis of protein levels through regulating the expression of multiple E3 ligase genes. Moreover, another large portion of this category contains genes involved in protein posttranslational modification such as genes encoding protein kinase and phosphatase (Data S5, S6), which concurs with the GO term *signal transduction* also being enriched in GNC‐ and CGA1‐associated genes (Fig. S5).

### 3.7 Transcription factors as targets of GNC and CGA1

GO and pathway analyses showed that transcription factors were overrepresented in the GNC‐ and CGA1‐associated genes (Figs S5, S6). About 8% of GNC‐associated genes and 10% of CGA1‐associated genes were assigned to the molecular function GO term *transcription factor activity* (Data S3, S5). Consistent with this, the cellular component GO term, the *nucleus,* and the biological process GO term *transcription, DNA‐dependent,* were highly enriched (Fig. S5). These include genes encoding AP2/ERF, bZIP, MYB, bHLH, and MADS‐box transcription factors, which might further regulate expression of genes involved in diverse processes. Some of these genes are known as key regulators of hormone signaling pathways. For example, several *Arabidopsis Response Factors* (*ARRs*), *Cytokinin Response Factors* (*CRFs*), and *AP2/ERFs* factors involved in cytokinin signaling were detected as GNC‐ or CGA1‐associated genes (Data S2, S5). This is consistent with a previous report that GNC and CGA1 are involved in multiple aspects of cytokinin‐regulated development (Ranftl et al., 2016). The overrepresentation of diverse transcription factors as target genes suggests that GNC and CGA1 could function as upstream regulators of different pathways.

### 3.8 GNC and CGA1 regulate plant greening and development

**Figure 6:**
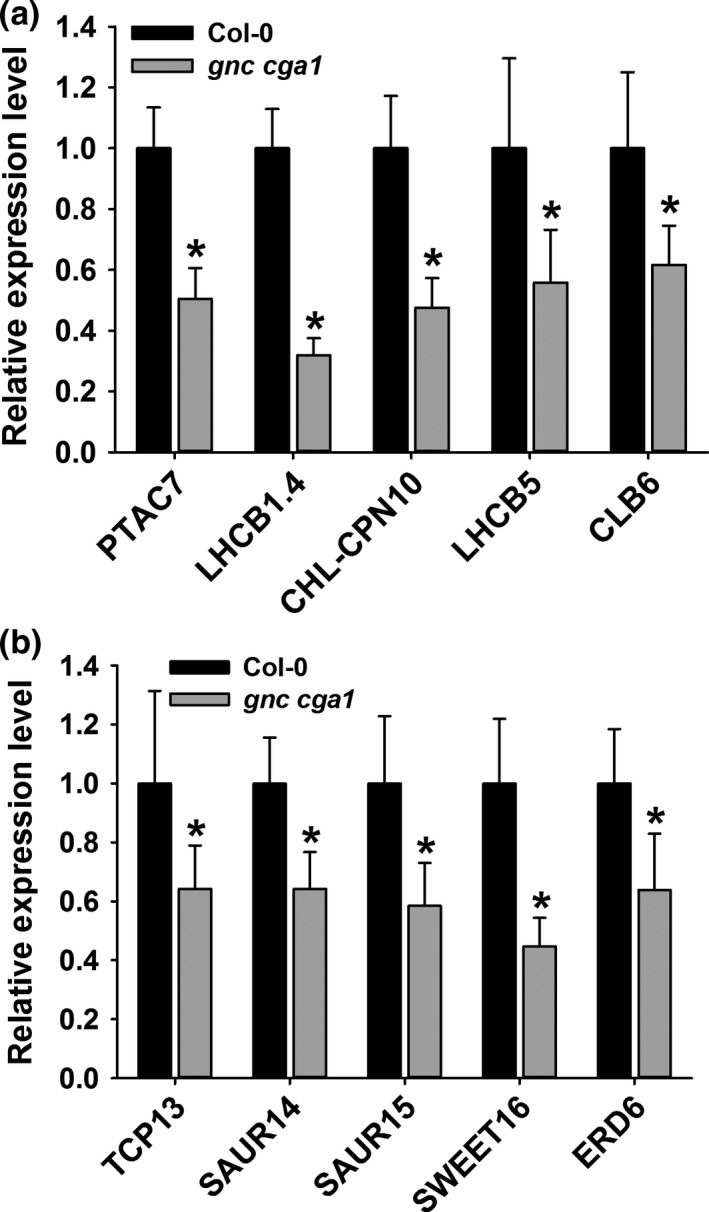
Gene expression analysis of selected target genes of GNC and CGA1. (a) Chlorophyll‐ and chloroplast‐related genes are down‐regulated in the *gnc cga1* double mutant. (b) Genes controlling development are down‐regulated in the *gnc cga1* double mutant. Statistical analysis was performed using the Student's *t* test (*p *<* *.01)

The most prominent defect in *gnc* as well as in the *gnc cga1* double mutants is the lack of chlorophyll whereas the *cga1* mutant only exhibits a weaker phenotype (Fig. S7). A previous report showed that chloroplast development, growth, and division were also impaired in the *gnc cga1* double mutant (Chiang et al., 2012). The cellular component GO term *chloroplast* was found only enriched in the GNC‐associated genes, but not in those of CGA1 (Fig. S5), suggesting that GNC has a more direct role in regulating the chloroplast‐related greening process, which is also consistent with the observed phenotype of the corresponding mutant (Fig. S7). Expression levels of some chloroplast or chlorophyll genes such as *PTAC7*,* CHL‐CPN10*,* LHCB1.4*,* LHCB5,* and *CLB6*, identified in the ChIP‐seq experiment (Data S2), were quantified and were found to be down‐regulated in the *gnc cga1* double mutant (Figure 6a). In addition, as GNC‐specific targets, expression of *PTAC7* and *LHCB1.4,* were down‐regulated in the *gnc* mutant (Fig. S8a), but not significantly changed in the *cga1* mutant (Fig. S8b). *CPSUFE*, another chloroplast‐related gene, was up‐regulated in the *cga1* mutant, which is not surprising given the fact that *GNC* was up‐regulated in the *cga1* mutant (Fig. S8a). Although CGA1, but not GNC, was detected to bind to *CLB6* and *CHL‐CPN10*, expression of *CBL6* was up‐regulated in the *cga1* mutant and expression of *CHL‐CPN10* was down‐regulated in the *gnc* mutant. These results suggest that besides direct transcriptional regulation, these two factors participate in other indirect regulatory pathways controlling gene expression.

In addition, as GNC and CGA1 also affect plant size, architecture, flowering, and senescence, we hypothesized that the GNC‐ or CGA1‐associated genes could be involved in plant developmental pathways. Interestingly, the GO term *developmental processes* was significantly enriched among the GNC‐ and CGA1‐associated genes compared to the frequency in the whole genome (Fig. S5). Two previously identified target genes of GNC or CGA1, *SOC1,* and *SPCH*, which, respectively, regulate flowering time and stomatal development in hypocotyls (Klermund et al., 2016; Richter et al., 2013a), were also detected in our results (Data S2). *SOC1* and *CRF2*, two common target genes of GNC and CGA1 identified in this study, were up‐regulated in both *gnc* and *cga1*. However, *SOC1* was up‐regulated in the *gnc cga1* double mutant, whereas the expression of *CRF2* was not significantly changed (Fig. S8c), which further suggests a complex cross‐regulation between these two factors. Among the transcription factors identified as GNC‐ or CGA1‐associated genes, several are involved in different aspects of plant development, such as NAC transcription factors, AGAMOUS‐like MADS‐box, TCP, and LBD factors (Data S2, S5). Other nontranscription factor proteins associated with plant development were also detected (Fig. S6; Data S2, S5). Expression levels of *TCP13*,* SAUR14*,* SAUR15*,* SWEET16,* and *ERD6*, which function in different developmental pathways were all decreased in the *gnc cga1* double mutant (Figure 6b), suggesting that these genes are targets of GNC or CGA1 in regulating plant development.

## 4 DISCUSSION

### 4.1 Identification of GNC and CGA1 binding‐associated genes reveals possible common and specific functions

The use of a ChIP‐seq approach to study the interactions between chromatin and proteins at a genomewide scale has contributed to the determination of the function and mechanisms of transcription factors (Song et al., 2016). Here, we applied this approach to analyze the binding events of GNC and CGA1 to broaden our understanding of their role in developmental processes. Among all the GNC or CGA1 binding peaks, about 30% of them were located within the 1 kb promoter region and most binding events were enriched in the first 200 bp directly upstream of the transcription start site (TSS) (Figures 2a,b, S1d). 1475 and 638 potential target genes of GNC and CGA1, respectively, were identified (Figure 3).

GNC and CGA1 have significant sequence similarity and function at least partially redundantly from a phenotypic point of view (Hudson et al., 2011; Mara & Irish, 2008; Reyes, Muro‐Pastor, & Florencio, 2004). In addition, co‐expression analysis shows a high percentage of convergence between the two genes (Aoki et al., 2016). However, the number of overlapping binding‐associated genes between GNC and CGA1 was extremely low (Figure 3a), indicating the possibility that most target genes are not shared by GNC and CGA1. These together suggest a cross‐regulation mechanism between GNC and CGA1, in which these two transcription factors regulate the expression of each other while each of them regulates a divergent set of genes. Evidence for this cross‐regulation was shown by ChIP‐qPCR with the binding of GNC to the *CGA1* promoter and the binding of CGA1 to the *GNC* promoter (Figure 3b,c). This would account for these genes having a high level of overlap in terms of co‐expressed genes, while still sharing few binding‐associated genes (Data S2, S6). This cross‐regulation was further supported by the induced expression of *GNC* and *CGA1* in their respective single‐mutant counterpart (Fig. S8a,b), as well as the different expression of *CRF2* in single and double mutants (Fig. S8c). It is interesting to note that the two GATA transcription factors, HAN and HANL2, which also belong to the GATA3 family, have been reported to be able to form homo‐ or heterodimers with members of the GATA3 family and to cross‐regulate each other (Zhang et al., 2013). In contrast, the low percentage overlap of GNC‐ and CGA1‐associated genes indicates that GNC and CGA1 are unlikely to regulate transcription through hetero‐dimerization, which is consistent with the evidence obtained from a yeast‐two‐hybrid assay (Behringer et al., 2014).

GO analysis of the GNC and CGA1 binding‐associated genes largely revealed the functional specificity of these two transcription factors (Figures 5a, S5). This was also consistent with the phenotypic divergence of the single mutants of *gnc* and *cga1*, in which the developmental defects were more significant in the *gnc* mutant than in *cga1* (Fig. S7).

### 4.2 The transcription factors GNC and CGA1 mediate complex genetic networks

Genomewide binding analysis of GNC and CGA1 reveals that they are involved in complex genetic networks, which is supported by the previous evidence showing that they both play roles in multiple processes (Behringer & Schwechheimer, 2015; Klermund et al., 2016; Ranftl et al., 2016). The binding‐associated genes of GNC and CGA1 are enriched in diverse pathways such as protein binding, modification and degradation, transcriptional regulation, signal transduction as well as developmental processes (Figure 5, S5, S6). The enrichment of non‐GATA/C motifs in GNC and CGA1 binding peaks (Figure 4) suggests that GNC and CGA1 may be forming protein complexes with other transcription factors to control specific biological processes.

Protein binding was found to be the most enriched process in the GNC‐ and CGA1‐associated genes compared to other pathways, and most of the target genes found in this category were E3 ligases (Figure 5, S5–S6, Data [Link], [Link], [Link]). An E3 ligase gene, *CNI1*, which functions in carbon/nitrogen response like GNC, was identified as a GNC‐associated gene (Bi et al., 2005), suggesting that GNC may regulate nitrogen response and carbon metabolism through *CNI1*. Previous studies revealed that GNC and CGA1 protein are unstable proteins which can be degraded through the ubiquitin‐proteasome system (UPS) (Behringer et al., 2014; Richter et al., 2013b). However, little is known about the role of GNC and CGA1 or other GATA members in regulating the protein degradation pathway. In addition, most of the E3 ligase genes identified in this study have not been well characterized. This opens opportunities for further studies in exploring the target genes of GATA transcription factors that are involved in the UPS system.

In our ChIP‐seq dataset, ~10% of GNC‐ or CGA1‐associated genes encode transcription factors (Fig. S5; Data S6). An interesting example of their potential role in pathway regulation involves the cytokinin signaling pathway. Recently, the cytokinin response factors CRF1, CRF2, CRF3, CRF5, and CRF6 were reported to be functionally redundant positive regulators of early senescence. The over‐expressors of these genes showed early senescence and the *crf1, 3, 5, 6* quadruple mutant displayed significantly delayed senescence when compared to WT (Raines et al., 2016). Interestingly, some genes encoding *CRFs* and *ARRs* were identified in the ChIP‐seq datasets (Data S2). The early senescence phenotype in the *gnc cga1* double mutant suggests the possibility that GNC or CGA1 regulate these key regulators in the cytokinin signaling pathways in controlling the senescence process. GNC and CGA1 as well as other members of the LLM‐domain containing B‐GATA factors also function redundantly in different aspects of cytokinin‐regulated development (Ranftl et al., 2016). Given the previous evidence that GNC and CGA1 are also involved in GA and auxin signaling (Richter et al., 2010, 2013b), it is possible that GNC and CGA1 function in multiple phytohormone signaling pathways. Some of the targets transcriptionally regulate diverse aspects of plant development, such as NAC factors (Kim, Nam, & Lim, 2016), AGAMOUS‐like MADS‐box factors (Ng & Yanofsky, 2001), TCP factors (Danisman et al., 2013; Martín‐Trillo & Cubas, 2010), and LBD factors (Husbands, Bell, Shuai, Smith, & Springer, 2007) (Data S2, S5). Interestingly, some of the non‐GATA/C motifs harbored in the binding peaks of GNC and CGA1 are consensus binding sites for some of these transcription factors. For example, TCP factors bind the PCF motif (Lu et al., 2013) and E‐box (G‐box) motif is the specific binding site of bHLH transcription factors (Toledo‐Ortiz, Huq, & Quail, 2003) which can interact with LBD proteins (Husbands et al., 2007). Additional work is needed to decipher the complexity of multiple transcription factors regulating these genes.

Expression analysis of target genes in the *gnc* and *cga1* single mutants as well as in the *gnc cga1* double mutant also revealed a complex regulatory mechanism. *CHL‐CPN10* and *SAUR70* were only detected in the CGA1 ChIP‐seq dataset although their expression levels were also altered in *gnc*. *CLB6*, a *CGA1* target, was up‐regulated in *cga1* (Fig. S8a,b). This indicates that besides a cross‐regulation between GNC and CGA1, other types of mechanisms controlling gene expression are also involved. In addition, the expression of *SOC1*, a common target of GNC and CGA1, was up‐regulated in both single mutants as well as in the double mutant. Although in our assay GNC and CGA1 only displayed transcription activation activity, the above observations suggest that these factors also function as repressors in some specific developmental processes and signaling pathways.

In conclusion, this study characterized the possible downstream target genes of *Arabidopsis GNC* or *CGA1,* revealing common and specific roles in multiple processes of plant growth and development. GNC and CGA1 have been shown to be involved in many important processes including greening, flowering time, senescence, plant architecture and are key components of hormone signaling pathways. The downstream target genes identified help considerably in understanding the mechanisms via which this regulation occurs. Further, we present evidence that GNC and CGA1 cross‐regulate each other which accounts for their overlapping and diverse physiological roles in the *Arabidopsis* genome.

## CONFLICT OF INTEREST

The authors declare that there is no conflict of interest.

## AUTHOR CONTRIBUTIONS

X.Z., B.Y‐M., and S.J.R. designed the experiments. X.Z. conducted the experiments, analyzed the data, and wrote the manuscript. C.J., B.Y‐M., and S.J.R. revised the manuscript.
