# Supplementary material for: Genome‐wide binding analysis of AtGNC and AtCGA1 demonstrates their cross‐regulation and common and specific functions
Source: Plant Direct. 2017 Oct 16;1(4):e00016. doi: 10.1002/pld3.16 (PMC6508505; doi:10.1002/pld3.16)
Supplement: Supplementary file 1 [file PLD3-1-e00016-s001.pdf]

Supplemental Data

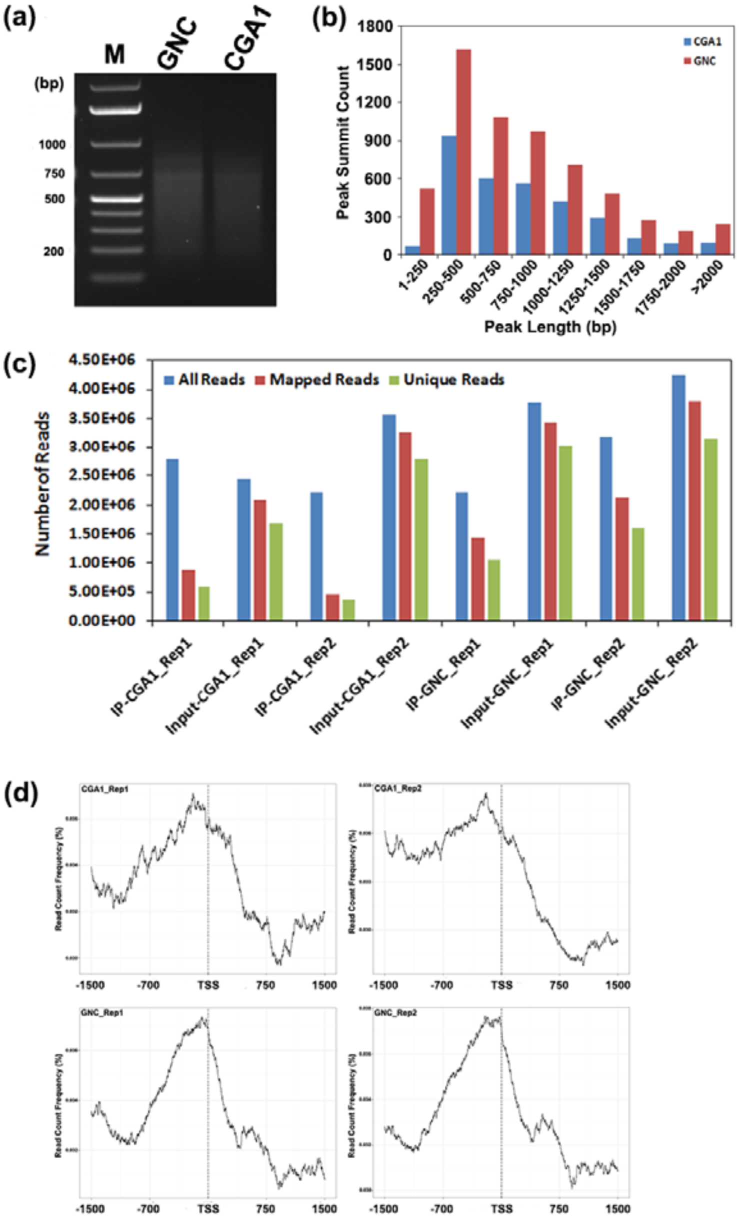

Figure S1. Overview of ChIP assay and ChIP-seq data.

(a) Sonicated chromatin from *Arabidopsis myc*-GNC and *myc*-CGA1 transgenic lines for the preparation of ChIP-DNA. (b) Peak length distribution of GNC and CGA1 ChIP-seq. (c) Sequencing reads of all the samples. (d) Peak distribution frequency around the transcription start site (TSS).

(a)

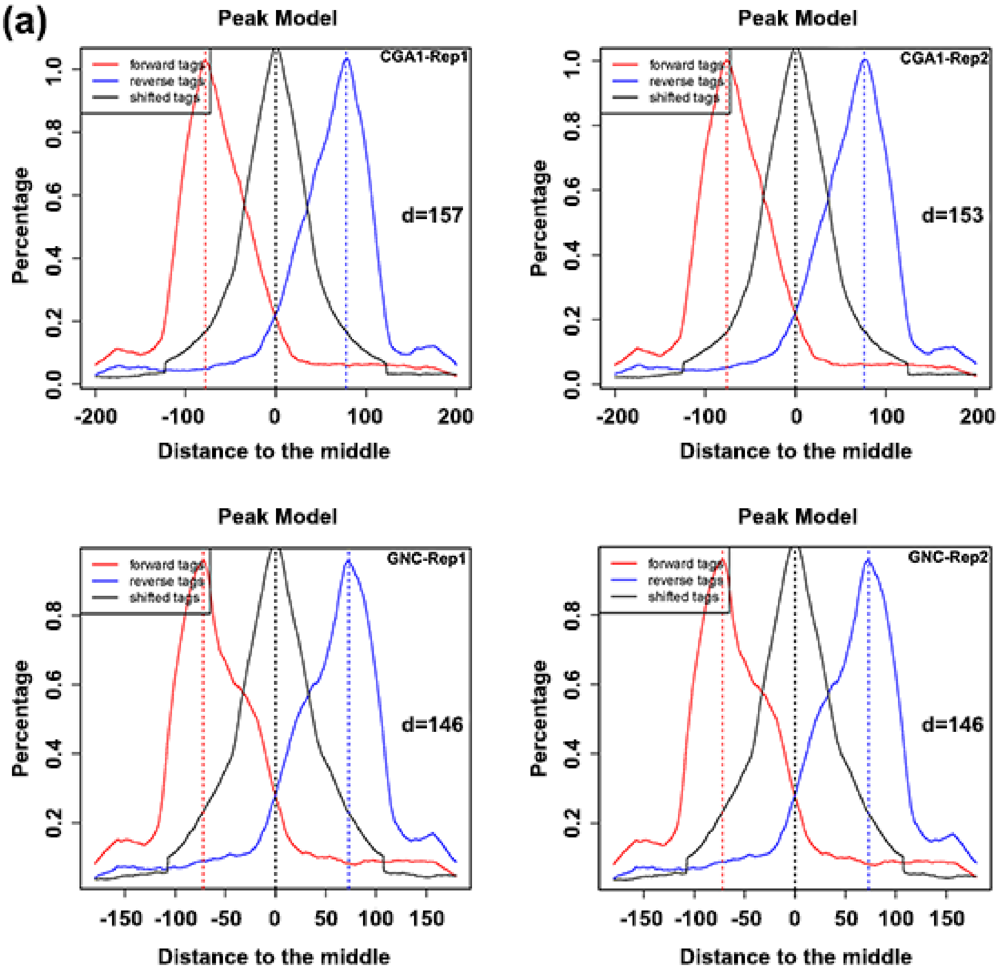

(b)

Sample	Shift Size	Read Length
CGA1_Rep1	79bp	50nt
CGA1_Rep2	77bp	50nt
GNC_Rep1	73bp	50nt
GNC_Rep2	73bp	50nt

Figure S2. Peak calling for GNC and CGA1 ChIP-seq data.

(a) Peak shift model for both replicates of GNC and CGA1 ChIP-seq data. (b) Peak shift size for constructing the peak model of GNC and CGA1 ChIP-seq data.

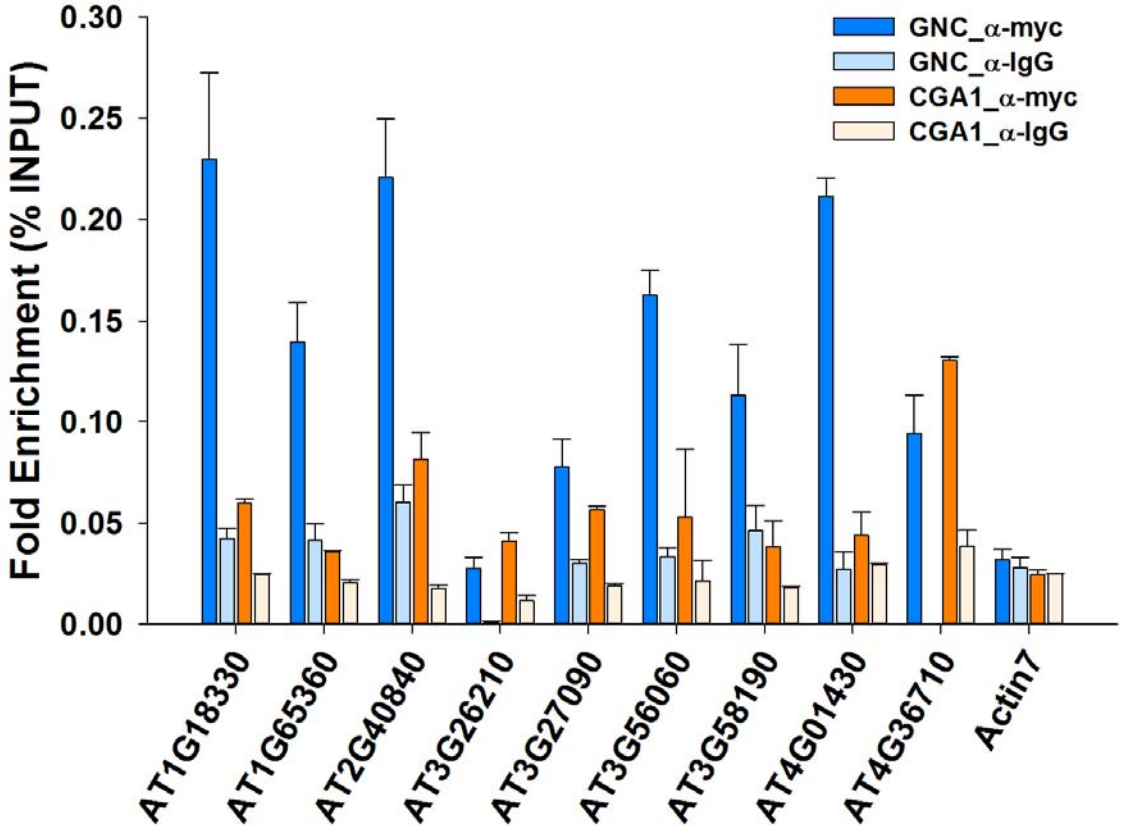

Figure S3. ChIP-qPCR verification of ChIP-seq data.

ChIP-qPCR verification of GNC and CGA1 binding sites identified in the ChIP-seq assay. The fold enrichment was normalized against the INPUT sample and expressed as % INPUT. Promoter of *Arabidopsis Actin7* gene was used as negative controls for ChIP-qPCR assay. α-*myc*, anti-*myc* antibody that used for immunoprecipitating GNC or CGA1 binding DNA; α-IgG, anti-IgG antibody that used as negative control for the ChIP assay. Value represents mean ± SD (n=3).

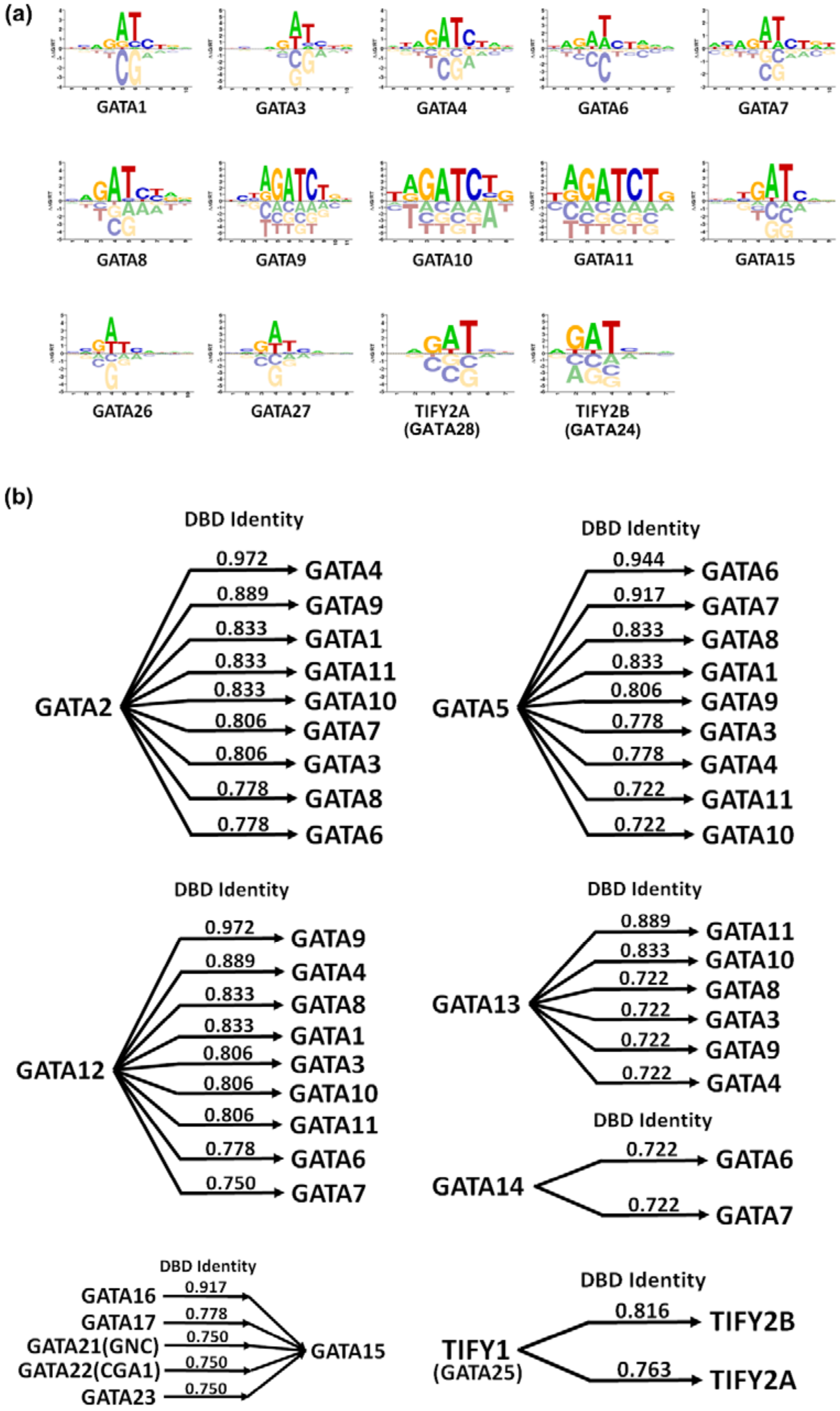
(a)
GATA1
GATA3
GATA4
GATA6
GATA7
GATA8
GATA9
GATA10
GATA11
GATA15
GATA26
GATA27
TIFY2A (GATA28)
TIFY2B (GATA24)
(b)
DBD Identity
GATA2
0.972 GATA4
0.889 GATA9
0.833 GATA1
0.833 GATA11
0.833 GATA10
0.806 GATA7
0.806 GATA3
0.778 GATA8
0.778 GATA6
DBD Identity
GATA5
0.944 GATA6
0.917 GATA7
0.833 GATA8
0.833 GATA1
0.806 GATA9
0.778 GATA3
0.778 GATA4
0.722 GATA11
0.722 GATA10
DBD Identity
GATA12
0.972 GATA9
0.889 GATA4
0.833 GATA8
0.833 GATA1
0.806 GATA3
0.806 GATA10
0.806 GATA11
0.778 GATA6
0.750 GATA7
DBD Identity
GATA13
0.889 GATA11
0.833 GATA10
0.722 GATA8
0.722 GATA3
0.722 GATA9
0.722 GATA4
DBD Identity
GATA14
0.722 GATA6
0.722 GATA7
DBD Identity
GATA16 0.917
GATA17 0.778
GATA21(GNC) 0.750
GATA22(CGA1) 0.750
GATA23 0.750
GATA15
DBD Identity
TIFY1 (GATA25)
0.816 TIFY2B
0.763 TIFY2A

Figure S4. Motifs of *Arabidopsis* GATA family members.

The *cis*-motifs of *Arabidopsis* GATA family were retrieved from the CIS-BP database (http://cisbp.ccbr.utoronto.ca/index.php).

(a) The motifs for the Arabidopsis GATA members with direct evidence from a protein binding microarray experiment. (b) Inferred motifs for the rest of the Arabidopsis GATA members based on the similarity of the DNA binding domain (DBD). DBD identity was applied to evaluate the likelihood of an inferred motif to the evidenced motif. For this family, transcription factors with DBD identity >= 0.7 will likely have a similar motif.

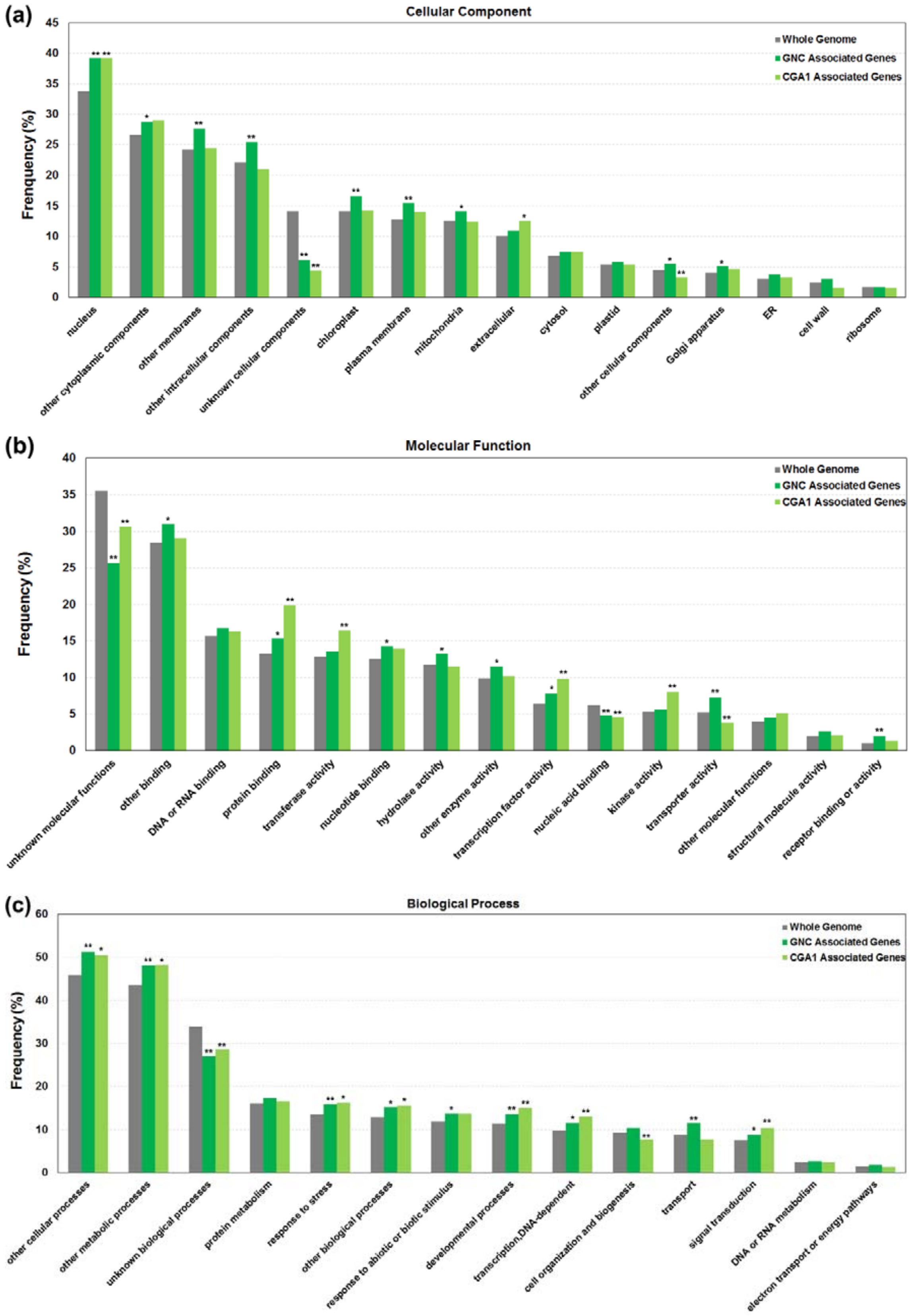
(a)
Cellular Component
Frenquency (%)
Whole Genome
GNC Associated Genes
CGA1 Associated Genes
nucleus
other cytoplasmic components
other membranes
other intracellular components
unknown cellular components
chloroplast
plasma membrane
mitochondria
extracellular
cytosol
plastid
other cellular components
Golgi apparatus
ER
cell wall
ribosome
(b)
Molecular Function
Frequency (%)
Whole Genome
GNC Associated Genes
CGA1 Associated Genes
unknown molecular functions
other binding
DNA or RNA binding
protein binding
transferase activity
nucleotide binding
hydrolase activity
other enzyme activity
transcription factor activity
nucleic acid binding
kinase activity
transporter activity
other molecular functions
structural molecule activity
receptor binding or activity
(c)
Biological Process
Frequency (%)
Whole Genome
GNC Associated Genes
CGA1 Associated Genes
other cellular processes
other metabolic processes
unknown biological processes
protein metabolism
response to stress
other biological processes
response to abiotic or biotic stimulus
developmental processes
transcription,DNA-dependent
cell organization and biogenesis
transport
signal transduction
DNA or RNA metabolism
electron transport or energy pathways

Figure S5. GO analysis of GNC and CGA1 binding associated genes.

The GO tool on the TAIR web page was used for gene ontology searches. For different datasets, frequency of each GO term under the main GO categories Cellular Component (a), Molecular Function (b) and Biological Process (c) were calculated. The counts under each GO term were expressed as frequencies of total counts in the whole genome. Stars represented significant enrichment or under-representation by hypergeometric test ($P < 0.001$).

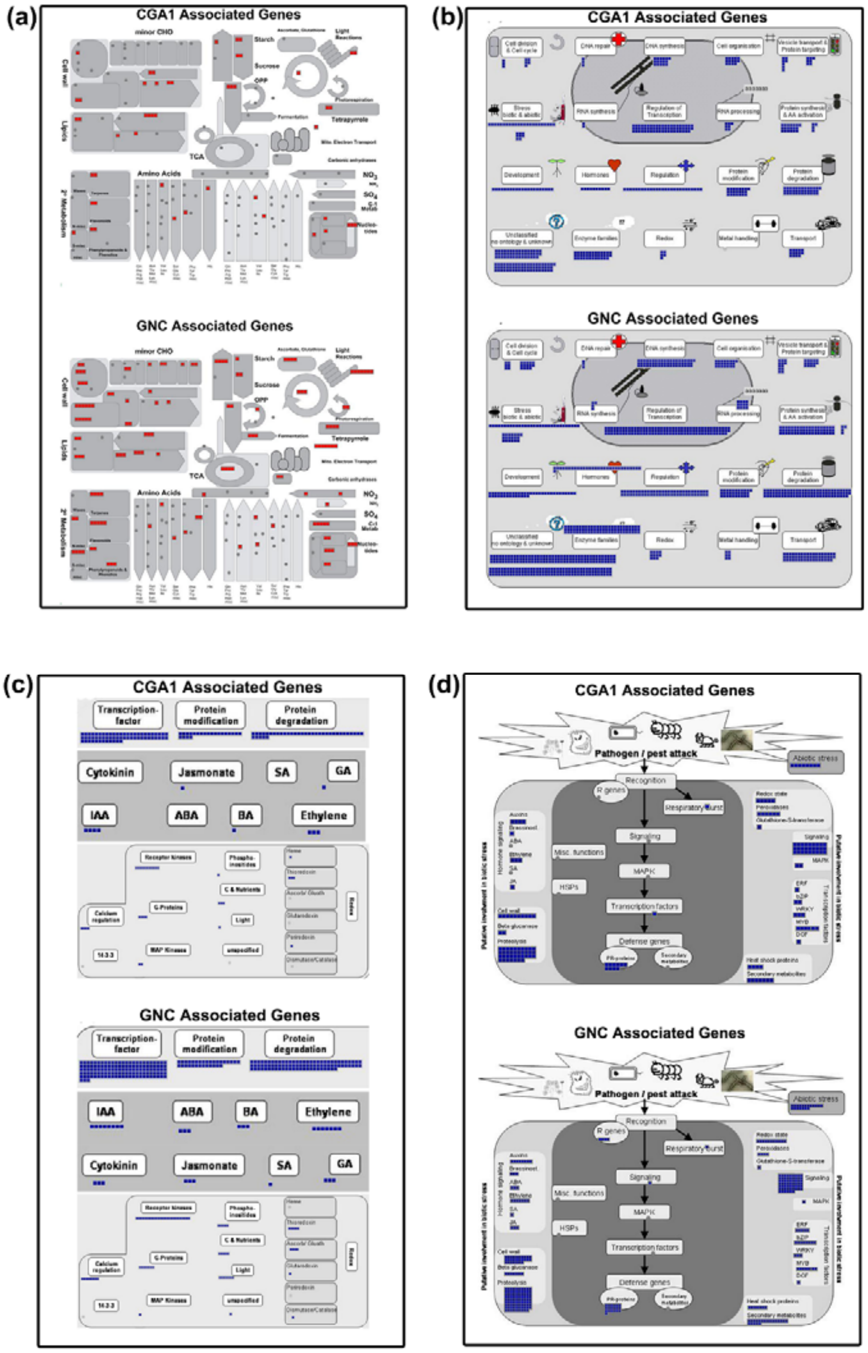

Figure S6. Functional pathway enrichment of GNC and CGA1 associated genes. Pathway analyses of GNC and CGA1 associated genes using MapMan showed the enrichment of GNC and CGA1 target genes in (a) metabolic, (b) cellular function, (c) regulation and (d) biotic stress pathways.

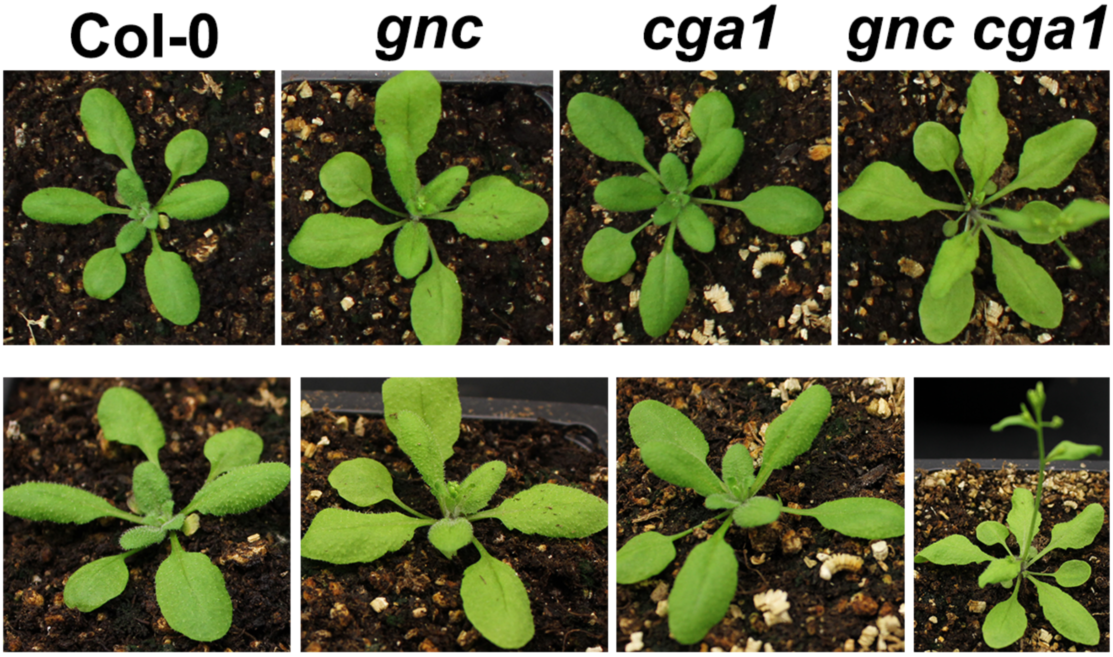

Figure S7. Phenotype of *Arabidopsis gnc*, *cga1* single and double mutants. Phenotype of 3-week-old *Arabidopsis* Col-0 wild-type, *gnc*, *cga1* and *gnc cga1* mutants showing the difference in greening and flowering time.

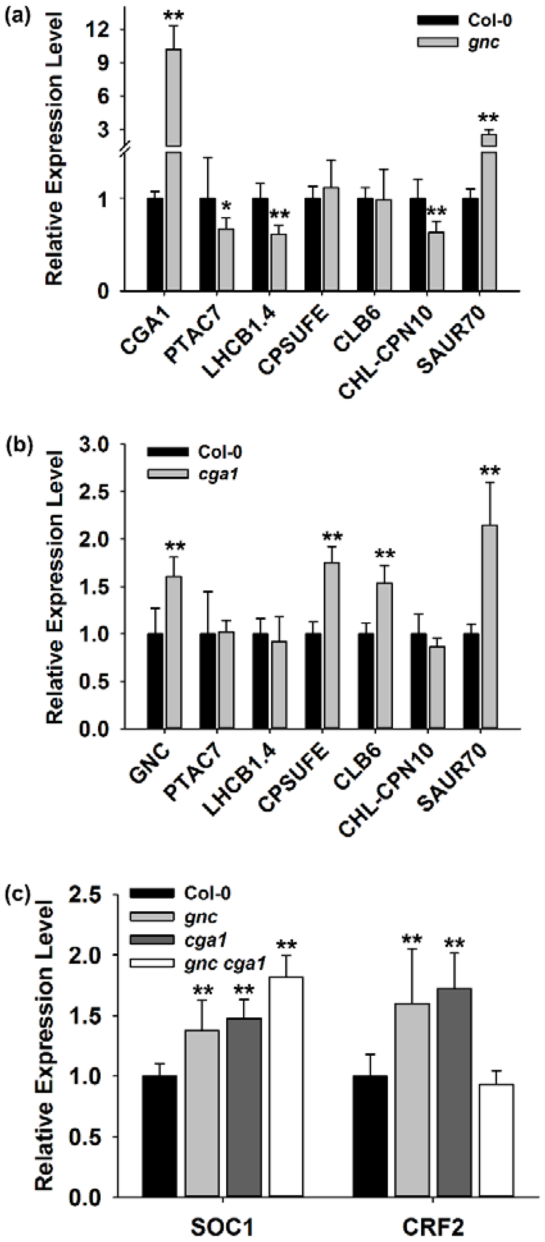

Figure S8. Expression analysis of GNC and(or) CGA1 ChIP targets in *gnc* and *cga1* mutants.

Expression of GNC or CGA1 targets in the (a) *gnc* and (b) *cga1* mutant. (c) Expression of common targets of GNC and CGA1 in the *gnc* and *cga1* single and double mutants. Statistical analysis was performed using the Student's *t*-test (* $P < 0.05$, ** $P < 0.01$)

Table S1. Distribution of GATA/C motif locations in the Arabidopsis Genome

Motifs	Numbers in Genome	Average genome length (bp) per motif
GATA (+ strand)	524137	One motif / 288 bp
TATC (- strand)	523698	One motif / 288 bp
GATC	435331	One motif / 274 bp

Table S2. Primer used in this study

Primer name	Sequence	Description
AtGNC CDS_F_ApaI	CCGGGCCCATGGATTCAAATTTTCATTACTCG	Transactivation assay
AtGNC CDS_R_KpnI	CGGGGTACCTCAACCGTGAACCATTCCATAC	
AtCGA1 CDS_F_ApaI	CCGGGCCCATGGGTTCCAATTTTCATTACAC	
AtCGA1 CDS_R_KpnI	CGGGGTACCTCACCCGTGAACCATTCCGT	
Renilla LUC_F_SmaI	TCCCCCGGGATGACTTCGAAAGTTTATGATCCAG	
Renilla LUC_R_NotI	ATAAGAATGCGGCCGCTTATTGTTCATTTTTGAGAACTCG	
GNC_ChIP_I_F	ATAGAGACCATGTGTGTTTG	ChIP-qPCR for CGA1 binding on GNC promoters
GNC_ChIP_I_R	GCTAAGATGAGAGATTGAGAG	
GNC_ChIP_II_F	CATTTCAGTTATTTATTTGTATCC	
GNC_ChIP_II_R	CTCAAATGACCATAAAGTATT	
GNC_ChIP_III_F	AATAGAGCCTTGATGAGATC	
GNC_ChIP_III_R	TATTGGAGAGTGAGAGCC	
CGA1_ChIP_I_F	TTCTGCTCGTGGTCTATA	ChIP-qPCR for GNC binding on CGA1 promoters
CGA1_ChIP_I_R	CGATGAAGCACAAAAGAGA	
CGA1_ChIP_II_F	TGCTCTTCATCAGTCTCTAA	
CGA1_ChIP_II_R	GTGTCTTGCTCACTAACAA	
CGA1_ChIP_III_F	CAGTTTGACAAGGGCGATA	
CGA1_ChIP_III_R	GGTAACGGATAGATTATAGACAGC	
AT1G18330_ChIP_F	CCCATTATCCCAACCATTT	ChIP-qPCR primer for validating the ChIP-seq data
AT1G18330_ChIP_R	CTCATTGTTCAGTTTTGTAAGT	
AT1G65360_ChIP_F	GTCGTCATGTGTCATGTTTA	
AT1G65360_ChIP_R	TTAATCGCCTGCTTTTGAAT	
AT2G40840_ChIP_F	GGAGAACTGTATTTGTTACTGTA	

AT2G40840_ChIP_R	GTATTGAAGAAGATCACGAGAT	
AT3G26210_ChIP_F	CGATCTCATCTGTTGTTCC	
AT3G26210_ChIP_R	GGTAATAGTCAAGCCATTCAT	
AT3G27090_ChIP_F	CTGTCTACTGAGGCAATGA	
AT3G27090_ChIP_R	GGTTGGTTGGATCTGTGA	
AT3G56060_ChIP_F	CAGCATCAATAGTCAATAGCC	
AT3G56060_ChIP_R	GGAATCCAAGTGGTCAAGA	
AT3G58190_ChIP_F	AGACCATTAAGCAGACAAGA	
AT3G58190_ChIP_R	TTACAAGCACCACAAGGA	
AT4G01430_ChIP_F	GAGTTGTTGTGCGTTGTA	
AT4G01430_ChIP_R	TGATACCGACGTGTCTAC	
AT4G36710_ChIP_F	CTCCACATAAAGCCCAGT	
AT4G36710_ChIP_R	ACCTAGTTGTTAGTACCACAC	
AtActin7_ChIP_F	CGTTTCGCTTTCCTTAGTGTTAGCT	
AtActin7_ChIP_R	AGCGAACGGATCTAGAGACTCACCTTG	
AtPTAC7_F	TTCACCTGTTCTTCTCCAT	Expression analysis of target genes
AtPTAC7_R	ATCGTCCTTCTTCGTCAA	
AtLHCB1.4_F	AAGGTTGGCTATGTTCTCT	
AtLHCB1.4_R	GCATTGTTGTTGACTGGAT	
AtCHL-CPN10_F	GGCTAACCATACGCTTCT	
AtCHL-CPN10_R	GGCAACAATACTCCACCT	
AtLHCB5_F	TTCAGGGCAGTTTCAAGA	
AtLHCB5_R	GCTCATCGCTAGTCTCAG	
AtCLB6_F	CGCAAGTATGGAGTGGAA	
AtCLB6_R	TGAAGGTGAGAGGTGTTAC	
AtTCP13_F	GCATCTTCAACCATCATCA	
AtTCP13_R	GCGGCTAGTAGTAGTAGAC	
AtSAUR14_F	TAAGAAAGGCAGAGGAAGAG	
AtSAUR14_R	TGAAGCGAGAAGCAAGAT	
AtSAUR15_F	ATGGCGGTCTATGTAGGA	
AtSAUR15_R	TCTGAGATGTGACTGTGAAG	
AtSWEET16_F	ATGTTATATCGGTGCTTGTC	
AtSWEET16_R	ACCAGGTGTCACTATTCC	
AtERD6_F	ACATCCGTAATAGCCACAA	
AtERD6_R	CAGAACAAGAAGCCATTAGG	
AtUBC21_F	TCCTCTTAACTGCGACTCAGG	
AtUBC21_R	GCGAGGCGTGTATACATTTG	
