# Supplementary material for: Genome‐wide binding analysis of AtGNC and AtCGA1 demonstrates their cross‐regulation and common and specific functions
Source: Plant Direct. 2017 Oct 16;1(4):e00016. doi: 10.1002/pld3.16 (PMC6508505; doi:10.1002/pld3.16)
Supplement: Supplementary file 8 [file PLD3-1-e00016-s008.docx]

**Response Letter**

Reviewer #1 : 
Authors verified that GNC and CGA1 are cross-regulated the expression of each other, however, binding analysis was conducted in GNC- and CGA1-overexpression plants thereby limits the information to reveal the upstream and downstream relationships between two factors under normal, greening, senescence and hormone signaling conditions. Therefore, the response of GNC and CGA1 to greening, senescence and hormone signaling should be examined in gnc, cga1 and gnc cga1 mutants to clearly demonstrate the upstream and downstream correlation. 
We thank the reviewer for the suggestion. However, it is not quite clear what point the reviewer wanted to address when mentioning “response of GNC and CGA1 to greening, senescence and hormone signaling in *gnc*, *cag1* and *gnc cga1* double mutants.” The purpose of the study was to identify downstream targets. Clearly this can be extended to analyze different genes, but we opted to provide sufficient data to support our findings and conclusions. Further, there will be no expression of GNC/CGA in the double mutants.

We agreed that more evidence was needed for the cross-regulation of GNC and CGA1. In this regard, we are providing new data on the regulation of genes associated to greening, senescence and hormone signaling in single mutants (see new Figure S8). We added corresponding statements in the Results section “**Identification of GNC or CGA1 associated genes reveals that these two transcription factors are cross-regulated”** as follows: “Expression analysis revealed that CGA1 was up-regulated about 10-fold in the *gnc* mutant (Figure S8a) and GNC was up-regulated about 1.6-fold in the *cga1* mutant (Figure S8b), suggesting the existence of a more complex compensation mechanism between GNC and CGA1 in regulating gene expression.” And we added to the Results section “**GNC and CGA1 regulate plant greening and development**” as follows: “*SOC1* and *CRF2*, two common target genes of GNC and CGA1 identified in this study, were up-regulated in both *gnc* and *cga1*. However, *SOC1* was up-regulated in the *gnc cga1* double mutant, whereas the expression of *CRF2* was not significantly changed (Figure S8c), which further suggests a complex cross-regulation between these two factors.”

In addition, we added corresponding statements in the Discussion section “**Identification of GNC and CGA1 binding associated genes reveals possible common and specific functions**” as follows: “This cross-regulation was further supported by the induced expression of *GNC* and *CGA1* in their respective single mutant counterpart (Figure S8a,b), as well as the different expression of *CRF2* in single and double mutants (Figure S8c).” We also added the statements in the Discussion section “**The transcription factors GNC and CGA1 mediate complex genetic networks**” as follows: “Expression analysis of target genes in the *gnc* and *cga1* single mutants as well as in the *gnc cga1* double mutant also revealed a complex regulatory mechanism. *CHL-CPN10* and *SAUR70* were only detected in the CGA1 ChIP-seq dataset although their expression levels were also altered in *gnc*. *CLB6*, a *CGA1* target, was up-regulated in *cga1* (Figure S8a,b). This indicates that besides a cross-regulation between GNC and CGA1, other types of mechanisms controlling gene expression are also involved. In addition, the expression of *SOC1*, a common target of GNC and CGA1, was up-regulated in both single mutants as well as in the double mutant. Although in our assay GNC and CGA1 only displayed transcription activation activity, the above observations suggest that these factors also function as repressors in some specific developmental processes and signaling pathways.”

Figure 6 a,b compared the gene expression analysis of selected target genes of GNC and CGA1 between WT and gnc cga1 double mutants. However, this manuscript suggesting that GNC has a more direct role in regulating the chloroplast-related greening process, which is also consistent with the observed phenotype of the corresponding mutant. Therefore, I would suggest authors also examine the expression of selected target genes in gnc and cga1 single mutant background to clearly clarify the difference of selected target gene expression in different mutant background.

We thank the reviewer for the suggestion. We have provided extra data to address this question (Figure S8) and have added the statements in the Results “**GNC and CGA1 regulate plant greening and development**” section as follows: “In addition, as GNC-specific targets, expression of *PTAC7* and *LHCB1.4* were down-regulated in the *gnc* mutant (Figure S8a), but not significantly changed in the *cga1* mutant (Figure S8b). *CPSUFE*, another chloroplast related gene, was up-regulated in the *cga1* mutant, which is not surprising given the fact that *GNC* was up-regulated in the *cga1* mutant (Figure S8a). Although CGA1, but not GNC, was detected to bind to *CLB6* and *CHL-CPN10*, expression of *CBL6* was up-regulated in the *cga1* mutant and expression of *CHL-CPN10* was down-regulated in the *gnc* mutant. These results suggest that besides direct transcriptional regulation, these two factors participate in other indirect regulatory pathways controlling gene expression.”

In addition, there are several points that need clarification and/or further experiments, as detailed below. 
1. Page 9, (Yoo et al., 2007) should be changed to Yoo et al. (2007).

This was modified accordingly.

2. Page 17, numbers of genes bind to CGA1 (638) is not consistent with numbers shown in the Abstract (648).

It should be 638, the wrong number has been corrected.

3. Many references should be carefully checked. Several references contained more than one capitaized letters in the title. 
They have been modified as suggested.

Reviewer #2 : 
The analysis pipeline is well-described and the data are well-interpreted. My major concern is the quality of the ChIP-Seq data. From Fig S1c, it seems that ~60% of IP-GNC reads can be mapped to genome, while the mapping percentage for IP-CGA1 is less than 30%. For IP-CGA1_rep2, the unique reads is only ~15% of raw reads. The author need to figure out the reason: why majority of sequenced reads cannot be mapped to genome. 
We thank the reviewer for raising this concern. We did notice the variations in the mappability for different ChIP-seq runs. Low read mappability is not uncommon in this type of experiments.

In our efforts to substantiate the reason causing that, we want to point to a paper published on *Scientific Report* in 2015 (Ouma et. al., *Scientific Reports* (2015) 5, 8635. doi:10.1038/srep08635**)**. In that study, the authors obtained the reads of 193 ChIP-seq runs from different organisms including H. sapiens (72 runs), C. elegans (35 runs), D. melanogaster (19 runs), Z. mays (46 runs) and A. thaliana (21 runs). Similar variations in the mappability of the ChIP-seq reads were found across every organism, which varied from 20% to 90% of reads that failed to align to the corresponding reference genome. Especially in Z. mays, the uniquely mapped reads only account for 3% to 25% of all sequencing reads.

Therefore, a low read mappability seems to be an inevitable phenomenon in ChIP-seq experiments and no clear answer has been suggested so far to significantly reduce it.

Minor comment: 
1, It seems that GNC and CGA1 are not in the target gene list. As the authors already have ChIP-Seq data, it is better to show all the data (all 8 libraries) in GNC and CGA1 promoter to support the cross-regulation model.

We generated 8 ChIP-seq libraries, however 4 of them were INPUT libraries which were used as controls in the bioinformatics analysis to identify the GNC and CGA1 binding peaks. Therefore, after the peak calling, only 4 datasets were generated, two for GNC binding peaks and two for CGA1 binding peaks. We summarized these data in supplemental dataset S1, which contained the peaks identified in both replications of GNC and CGA1 ChIP assay. We have double checked the datasets and CGA1 (GNL) was detected in GNC-rep2 dataset and GNC was detected in CGA1-rep1 dataset.

2, Fig 2c, for peak screenshot, it is better to show all 8 libraries' data and show representative peaks of the three different groups: CGA1-specific, GNC- specific, and common peaks. The authors also should add y-axis to show the normalized depth of reads.

We thank the reviewer for the suggestion. Fig. 2c was revised as suggested.

3, For Fig4, the sequence logo figure should have y-axis as bits.

We thank the reviewer for the suggestion. We agree that usually motifs are presented as sequence logo with bits on Y-axis. However, we also noticed that the output of the motif search varies among different programs. We used ChIPSeek, which integrates the HOMER package for motif search. The motifs generated by the HOMER are in the format that we presented in Fig 4 with no Y-axis. We found other publications using this HOMER software and the format representations are similar. As example, Nagel, D.H. et, al., Genome-wide identification of CCA1 targets uncovers an expanded clock network in Arabidopsis. (2015) *Proc. Natl. Acad. Sci. USA*, 112(34): E4802-E4810. Therefore, we decided to keep Fig. 4 as is.

4, For Fig 6, the asterisk are labeled but no statistical test was provided in the legend

The method for statistical analysis was added in the legend of Fig. 6 and the corresponding Fig.6 with legend was modified.

5, In the manuscript (abstract part), the author said that the target gene numbers are 1475 and 648. However, in the Supplemental Table, it seems that the target gene numbers should be 1474 and 638. Please double-check this and make corrections.

The numbers were corrected both in text and in figure.

6, The authors should italicize "Arabidopsis" in the whole manuscript. Now some are italicized but some are not.

They were revised as suggested.

7, Line 605, "over-lapping" should be "overlapping". Also the period at the end of the line should be removed.

This was revised as suggested.

8, Line 30, give the abbreviation "ChIP-Seq" after "chromatin immunoprecipitation sequencing".

This was revised as suggested.

9, Line 187, change "ChIPed DNA" to "ChIP DNA"

This was revised as suggested.

10, Line 199, "Tair10" should be "TAIR10"

This was revised as suggested.

11, Line 320, 334, 513, 624, 628 and in figure legend, "1 Kb" should be "1 kb"

This was revised as suggested.
